# Levels of care for maternal and neonatal healthcare: a scoping review

**DOI:** 10.7189/jogh.16.04035

**Published:** 2026-03-20

**Authors:** Shaina Mackin, Louise Tina Day, Caity Dekker, Isabel Gouse, Michuki Maina, Jalemba Aluvaala, Allisyn Moran, Revisioning Emergency Obstetric and Newborn Care (EmONC) levels-of-care workstream, Allisyn Moran, Jalemba Aluvaala, Louise Tina Day, Shaina Mackin, Caity Dekker, Michuki Maina, Isabel Gouse, Patience Afulani, Patsy Bailey, Lynn Freedman, Tedbabe Hailegebriel, Elimase Kamanga, Mary Kinney, Ornella Lincetto, Samantha Lobis, Zoe Matthews, Jean-Pierre Monet, Isabelle Moreira, Sarah Moxon, Kristine Nilsen, Cynthia Boschi, Kate Ramsey, Jennifer Requejo, Sanam Roder-DeWan, Suzanne Stalls, Caitlin Warthin

**Affiliations:** 1 Averting Maternal Death and Disability, Columbia University Mailman School of Public Health, New York, New York, USA; 2 Department of Infectious Disease Epidemiology and International Health, London School of Hygiene & Tropical Medicine, UK; 3 KEMRI Wellcome Trust Research Programme, Nairobi, Kenya; 4 KEMRI Wellcome Trust Research Programme and Department of Paediatrics and Child Health, University of Nairobi, Nairobi, Kenya; 5 Sexual, Reproductive, Maternal, Child, Adolescent and Ageing Health Department, World Health Organization, Geneva, Switzerland

## Abstract

**Background:**

To meet the 2030 aims of the Global Strategy for Women’s, Children’s and Adolescents’ Health and Sustainable Development Goals, annual rates of maternal and newborn mortality and stillbirth must decrease. The organisation of maternal and newborn health (MNH) services influences access to and quality of care. We designed this scoping review to understand how levels of MNH care are organised in different country contexts.

**Methods:**

We conducted a scoping review of peer-reviewed literature published after 2009. Based on the World Health Organization (WHO) quality-of-care framework, we conducted descriptive and deductive textual narrative analysis to identify the reported number of levels of MNH care stratified by country and mortality rates; describe how levels are conceptualised; and explore alignment of levels for the maternal-newborn dyad.

**Results:**

We included 162 of 3591 reports. The number of MNH facility levels of care across 56 countries ranged from two to seven. Types of identified MNH care facilities were described at varying levels. Two levels of care were reported in 5% of cases, three levels in 55%, four levels in 30%, five levels in 8%, and six and seven levels in <1% of cases. Home and community-based MNH care (non-facility) was reported in 8% of country descriptions. Countries with the lowest stillbirth, maternal, and newborn mortality rates mostly reported three or four facility levels. The criteria used to distinguish MNH levels of care as low, intermediate, and high were aligned with domains of the WHO quality-of-care framework, mostly human and physical resources.

**Conclusions:**

Levels of MNH care described in the literature were distinguished by characteristics, including provision and experience of routine and emergency care. Three or four levels of MNH facility care were most commonly reported. Linking maternal and newborn facility care to community connections is an important consideration to ensure equitable access to routine and emergency care.

Increasing access to institutional childbirth has been a major strategy for several decades to end preventable maternal and newborn deaths and stillbirths. Although >80% of global births currently occur in health facilities [1], mortality rates are decreasing too slowly to meet the Global Strategy for Women’s, Children’s and Adolescents’ Health and the Sustainable Development Goals [2].

The organisation of the maternal and newborn health (MNH) care affects women’s and newborns’ access to care, and currently, access to MNH services remains inequitable worldwide. Emergency clinical interventions are necessary to prevent death for the most common direct causes of death and disability for both women (*e.g.* haemorrhage, eclampsia, and sepsis) and newborns (*e.g.* intrapartum-related complications, sepsis, and preterm birth) [3–5]. The emergency obstetric care (EmOC) framework, used since 1997 in high-mortality/morbidity settings to plan the availability and accessibility of emergency interventions, is currently being revised [6–9]. The existing framework conceptualises two levels of EmOC health facilities based on the reported performance of tracer ‘signal functions,’ or emergency clinical interventions, within a specified timeframe. These two levels are comprehensive EmOC (CEmOC), in which all nine signal functions are performed, and basic EmOC (BEmOC), including a subset of seven signal functions [8]. In 2012, researchers proposed measuring a set of newborn-specific ‘signal functions’ across different levels of emergency care [10]. In 2018, the World Health Organization (WHO) and United Nations Children’s Fund (UNICEF) proposed levels of care for small and sick newborns, also based on clinical intervention availability [11].

The organisation of MNH care is essential to delivering high-quality services, and the poor quality of care is a major contributor to preventable deaths of women, newborns, and stillbirths [12]. The 2015 WHO vision of quality of care for MNH articulates the structures and processes needed for people-centred health outcomes, including the provision and experience of care [13,14]. The resulting framework articulates eight interconnected domains: evidence-based practices, actionable information systems, functional referral systems, effective communication, respect and dignity, emotional support, competent and motivated human resources, and essential physical resources.

Health service delivery is typically organised into levels that vary by setting – primary, secondary, and tertiary. Improving MNH care services organisation is an important lever for change to accelerate progress in ending preventable mortality and morbidity among women and children. As illustrated by the launch of the ‘Every Woman Every Newborn Everywhere’ platform, this includes a focus on aligning and integrating maternal and newborn service delivery to end preventable mortality and morbidity [15]. Recent WHO guidance on networks of care for MNH describes the importance of connections between levels of MNH care [16]. However, little is known about the optimal levels of care in the health system for high-quality MNH care, both for routine care and for care when complications arise.

In this research, we aimed to explore how countries, since the 2009 EmOC revision, organised MNH care into levels. We collaboratively designed this study with the project Revisioning EmONC ‘Levels of Care’ workstream’s technical advisors [9]. We had three goals: to report how many levels of MNH care have been organised in different country health systems and stratify them by mortality rates, to describe the criteria used to define and conceptualise maternal and newborn levels of care within country health systems, and to explore alignment of levels of care for the maternal-newborn dyad (**Table 1**).

**Table 1:** Objectives and research questions

Objectives and research questions
To report how many levels of MNH care are operationalised in country health systems
*How many levels of MNH care are operating in different countries’ health systems?*
To describe the criteria used to define and conceptualise MNH levels of care within country health systems
*How have experts defined levels of MNH care?*
*How have health systems organised levels of MNH care?*
*How does regionalisation fit into the definition and organisation of levels of MNH care?*
*How do referrals fit into the definition and organisation of levels of MNH care?*
*Can the domains of the WHO’s framework for the quality of MNH care be applied to defining and organising levels of MNH care?*
To explore the alignment of levels of care for the maternal-newborn dyad
*How have health systems and experts conceptualised the maternal-newborn dyad in the context of levels of care?*
To list measures identified to determine levels of MNH care
*What indicators do health systems and experts use for maternal and newborn levels of care?*

MNH – maternal and newborn health, WHO – World Health Organization

## METHODS

We designed this scoping review using the updated Arksey and O’Malley framework and reported its findings in accordance with the PRISMA-ScR [17–21] (Checklist S1 in the **Online Supplementary Documen**t).

### Search strategy and eligibility criteria

We reviewed peer-reviewed publications across all income settings to provide broad evidence on maternal and newborn levels of care. In October 2021, we searched four bibliographic databases – MEDLINE, Embase, Cochrane, and Google Scholar – using a search strategy designed collaboratively by the Revisioning EmONC project’s working group (Table S1 in the **Online Supplementary Document**). For feasibility, we limited Google Scholar results to the top 200 results. To ensure this did not lead to the omission of relevant literature, at the conclusion of title and abstract screening, from October 2021 to August 2022, we sent the list of included records to the Steering Committee of the Revisioning EmONC project and committee members to solicit feedback and identify potential studies that may have been omitted, based on their subject matter expertise. English, Spanish, Portuguese, French, and Arabic were included as eligible languages.

We used two search strings across all databases. Eligibility for the first string, which included MNH and levels of care keywords, was restricted to records published from 2009 onwards to reflect developments in the MNH levels of care literature since the last revision of the EmONC handbook in 2009 [8]. The second string combined two search terms from the 2020 WHO, United Nations Population Fund, and UNICEF review of maternal networks of care [22]. This search was restricted to publications after June 2020, when the landscape review was published, to identify updates in the literature (Table S2 in the **Online Supplementary Document**).

### Data management, extraction, and analysis

After identifying and removing duplicates, two primary reviewers (CD and SM) double-screened the remaining titles and abstracts. They initially calibrated their screening decisions by comparing and reaching consensus on a subset of records to ensure consistent application of the inclusion criteria and use of the data extraction tool (Table S3 in the **Online Supplementary Document**). Conflicts over whether records should be included or excluded were resolved by a third reviewer (LD). An additional reviewer with the necessary language proficiency (IG) screened Spanish records and then discussed with the primary reviewers (SM and CD) to decide on inclusion or exclusion. CD and SM conducted full text review and dual data extraction using a customised extraction tool in Covidence (Covidence, Melbourne, Victoria, Australia) for the following variables: study design, type of literature, units of analysis, client focus (*i.e.* maternal, neonatal), geography by WHO regions, number of MNH levels of care, descriptions of levels of care, discussion of the maternal-newborn dyad, and measures used to determine levels of care. Possible conflicts between reviewers (CD and SM) were resolved in consultation with a third researcher (LD).

We imported publicly available WHO/UNICEF data on maternal and neonatal mortality and stillbirths by country from the Healthy Newborn Network and merged them with extracted data to map MNH outcomes to the number of levels of care [23]. We then summarised, by outcomes, the number of MNH care levels operating in country settings. We categorised reports that described levels of care in country settings but did not explicitly state or describe how many levels existed as having an ‘unspecified’ number of levels.

**Figure 1:**
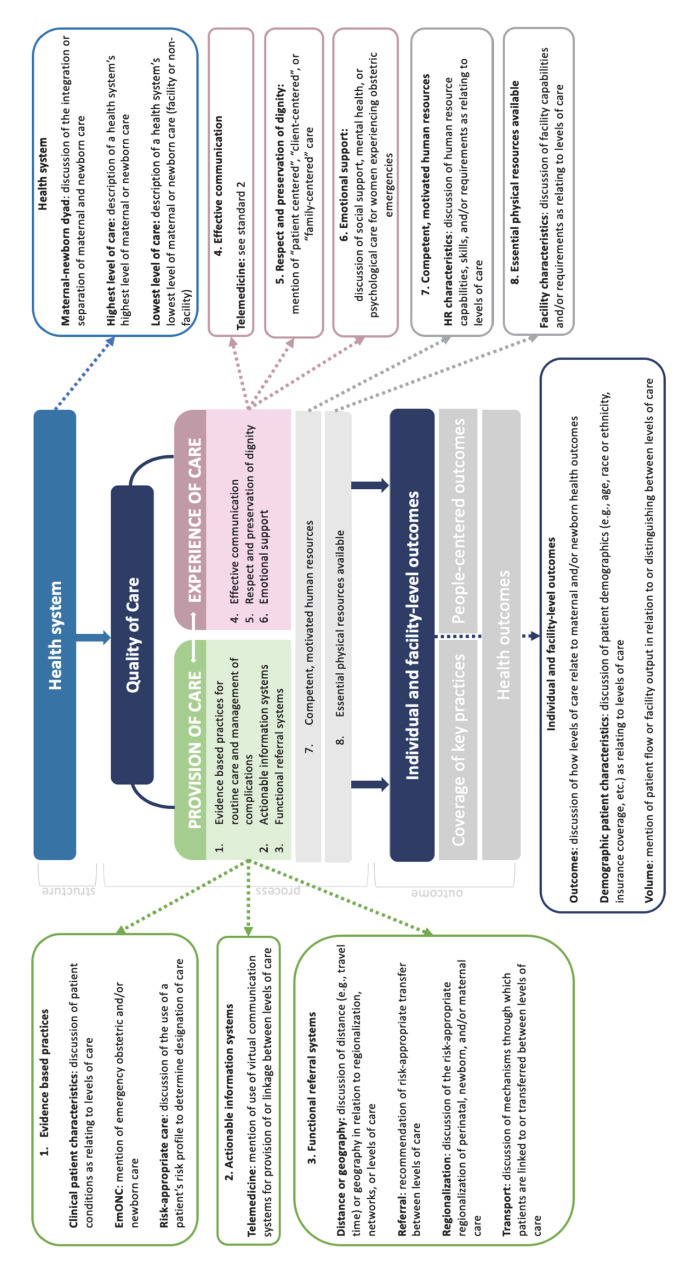
Results from textual narrative synthesis mapped to WHO framework for the quality of maternal and newborn health care [14].

We analysed qualitative data using textual narrative synthesis, conceptualising the criteria used for defining MNH levels of care. We primarily used a deductive analysis, with the domains of the WHO quality-of-care framework for MNH (modelled according to the Donabedian flow of structure, process, and outcome) as *a priori* nodes. We began with a codebook consisting of the framework’s domains. Following consensus among CD, SM, and LD, additional codes were introduced during textual narrative analysis as themes emerged from the literature. All codes mapped to the WHO framework for the quality of MNH care are presented in **Figure 1** [4,24,25]. Moreover, we generated descriptive summary statistics. The findings were discussed and reviewed by the Revisioning EmONC levels of care technical working group and Steering Committee to validate the relevance of the synthesised findings to strengthen credibility (Table S1 in the **Online Supplementary Document**).

We managed data using Zotero (Corporation for Digital Scholarship, Falls Church, Virginia, USA) reference manager for search results, Covidence (Covidence, Melbourne, Victoria, Australia) software for workflow management, *R*, version 4.1.1 (R Core Team, Vienna, Austria) for quantitative analysis, and NVivo, version 12 (Lumivero, Denver, Colorado, USA) for qualitative data.

## RESULTS

### Selection of sources and summary statistics

**Figure 2:**
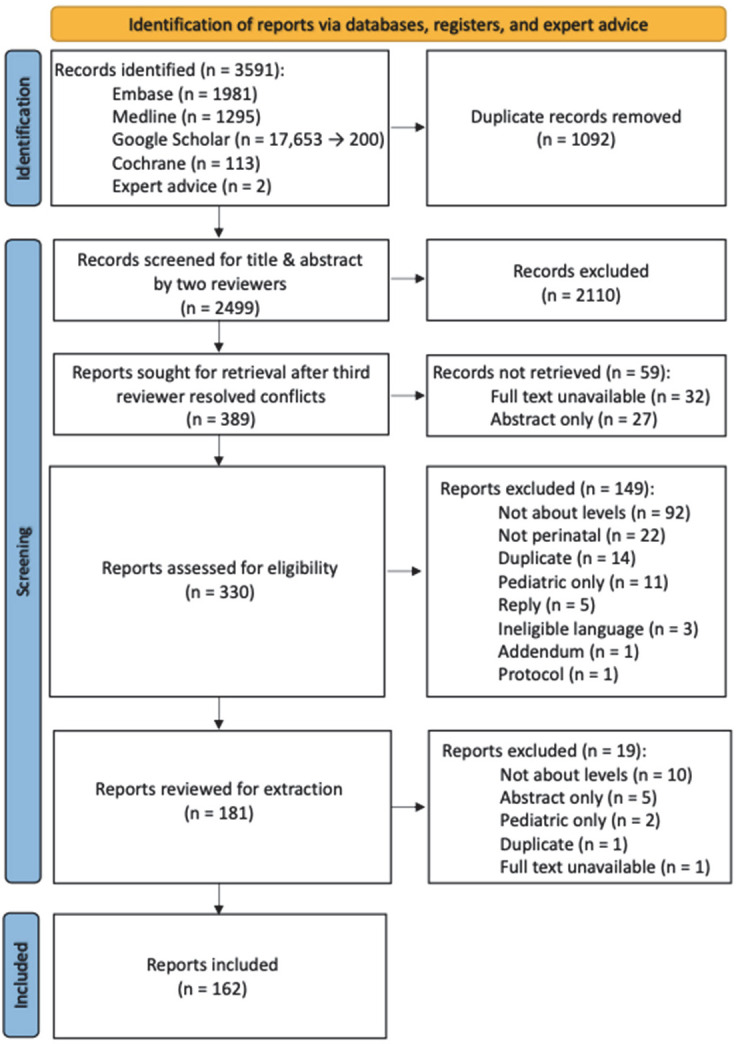
PRISMA flowchart of included reports. *For feasibility purposes, Google Scholar results were limited to the top 200 results.

We retained 389 of 2499 records for full-text review after title and abstract and retained 162 for data extraction (**Figure 2**; Table S4 in the **Online Supplementary Document**).

**Figure 3:**
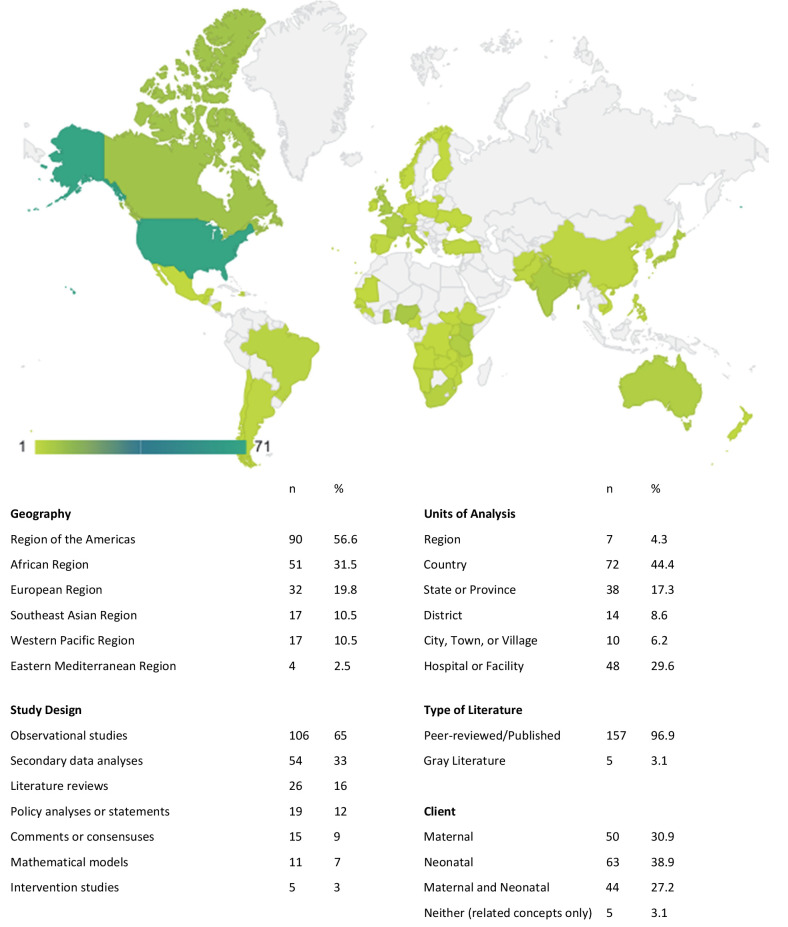
Characteristics of included reports regarding levels of care for maternal and neonatal healthcare (n = 162). The map presents the number of reviews per country.

More than half of the included reports were from the WHO Regions of the Americas (56%) and Africa (32%), with 89% of reports from the Americas reporting on the USA or Canada (**Figure 3**). The studies predominantly had observational (65%) and secondary analysis (33%) designs. The unit of analysis varied from multinational regions to countries to health facilities.

### Objective one: identifying the number of MNH levels of care and stratifying by mortality rates

Based on 121 (74.7%) of the 162 included reports, the number of MNH levels of care ranged between two and seven, with a median of four. The remaining 41 reports (25.3%) discussed concepts related to MNH levels of care without specifying a number. Reported MNH levels of care included ‘home’ (with or without provider presence), ‘community’ (unspecified whether at home or in basic facility), and ‘health facility’ levels (*e.g.* birthing centres, health posts, primary care facilities, hospitals, *etc.*). Quantifying the number of facility-based levels (excluding home and community-based ‘non-facility’ levels reported by studies), two, three, four, five, six, and seven levels of care were described in 8 (5.3%), 83 (54.97%), 46 (30.46%), 12 (7.95%), and 1 (<1%) of cases, respectively (Figure S1 and Table S5 in the **Online Supplementary Document**).

Different reports sometimes conflicted on the number of MNH levels of care described in the given countries. In ten out of 56 (17.9%) countries (Australia, Canada, Ghana, Nepal, South Africa, Spain, Tanzania, Turkey, the UK, and the USA), different reports reported different numbers of MNH levels. For example, Tanzania was said to have five levels by some reports, and three by others.

**Figure 4:**
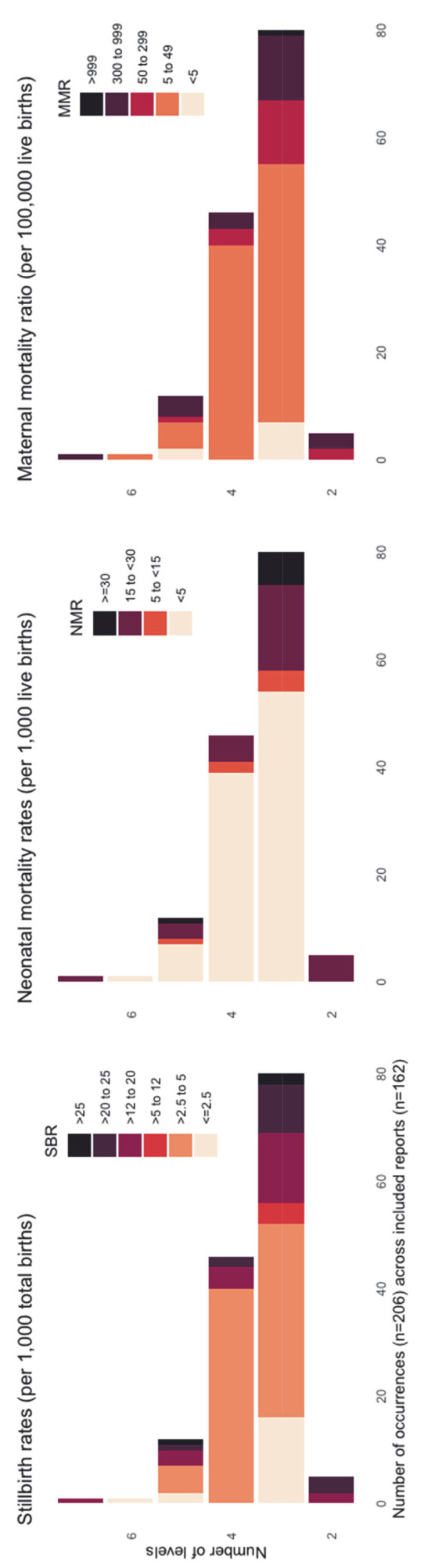
Number of MNH facility levels of care identified by outcomes.

Most of the lowest maternal, stillbirth, and neonatal mortality settings, including Denmark, Norway, Poland, Spain, and Italy, were described as having three levels of care (**Figure 4**; Table S6 in the **Online Supplementary Document**) [23].

### Objective two: defining and conceptualising MNH levels of care

Through the thematic analysis, we identified MNH levels of care as the lowest, intermediate, and highest levels of the health system. These levels had varied conceptualisations when mapped onto the structure, process, and outcome components of the WHO quality-of-care framework for maternal and newborn healthcare (**Figure 1**, **Table 2**) [14]. Some reports explicitly used the label ‘level 1’ for the lowest level of care and ‘level 2’, ‘level 3’, *etc*. for higher levels.

**Table 2:** Number of codes identified through thematic analysis mapped to the WHO quality-of-care framework among included papers (n = 162)

Codes	Occurrences	Papers
Health systems	18	13
*Maternal-newborn dyad*	56	31
*Highest level of care*	70	54
*Lowest level of care*	83	57
Domain 1: evidence-based practices	3	3
*Clinical patient characteristics*	110	71
*EmOC*	67	28
*Risk-appropriate care*	58	39
Domain 2: actionable information systems	4	3
*Telemedicine*	24	10
Domain 3: functional referral systems	23	17
*Distance or geography*	5	5
*Referral*	103	51
*Regionalisation*	162	78
*Transport*	75	39
Domain 4: effective communication	29	18
Domain 5: respect and preservation of dignity	8	7
Domain 6: emotional support	3	3
Domain 7: competent, motivated human resources	31	24
*HR characteristics*	110	72
Domain 8: essential physical resources available	21	17
*Facility characteristics*	157	83
Outcomes	64	38
*Demographic patient characteristics*	2	2
*Volume*	51	33

EmOC – emergency obstetric care, HR – human resources

### Structure

The level of MNH care was frequently categorised as low, intermediate, or high, though it was defined differently across settings. Most reports considered the lowest level (level 1) to be ‘health facilities’, typically falling below full hospitals and including primary health centres and community-based services. Examples included: community-based health planning and services and/or maternity homes (*e.g.* Ghana) [26–28], dispensaries (*e.g.* Tanzania) [29], community hospitals and/or birthing centres (*e.g.* USA and Canada) [30–34], and sub-base hospitals (*e.g.* Japan) [35].

From the included reports, 17 (10.5%) referenced non-facility (*e.g.* home or community) levels of MNH care at the lowest level of their health systems. In some of these cases, a three-level service delivery model was depicted: family and community (low level), outpatients and outreach clinics (intermediate level), and health facilities and hospitals (high level) [35]. Some reported that MNH care was structured as either home births or ‘level 1 facilities’, being considered the lowest level of care (*e.g.* UK) [36].

The category ‘high’ also varied in application across places of care, with some health systems using it to refer to any health facility or hospital, and others to specify a high-level health facility designation (*e.g.* tertiary) [35,37]. Occasionally, reports cited countries that consider primary health centres to be the highest level [38]. Further, 54 papers (33.3%) discussed ‘highest levels of care,’ while 57 papers (35.2%) discussed ‘lowest levels of care.’

### Process

We identified descriptors used to demarcate levels of care mapped to all the quality domains and dimensions of the quality-of-care framework [13,14].

### Provision of care

Evidence-based care (quality domain one) was used to delineate low, intermediate, and high categories of MNH services based on clinical conditions or risk-stratification [39]. Highest category services were conceptualised as care bundles for women and newborns with complications (*e.g.* blood transfusion and caesarean section for CEmOC) [40–44]. Some reports stratified the lowest level of care offered within their highest facility category as such (*e.g.* a ‘level I neonatal intensive care unit’) within an intensive care unit that also contains levels II–IV neonatal intensive care unit care) [45–51]. Further, 71 papers (43.8%) discussed evidence-based care in terms of ‘clinical patient characteristics.’

Lower-level categories of care referred to a range of service provision, including childbirth services with essential newborn care. One report from Kenya noted that the lowest levels may be the most neglected [39]. Another report describing Nepal’s health system stated that only the highest-level facilities have the capacity to offer even basic EmONC care [52]. One report proposed that established levels of intensive care be adapted for maternal care, with routine maternity care services at the lowest level [53].

Actionable information systems (quality domain two) were described in the reports in terms of the process linking levels of maternal and newborn care. Telemedicine emerged as a means of creating networks to improve the functioning of care levels. Examples were given from the start of the COVID-19 pandemic, showing how new telemedicine interfacility intensive care specialist consultation was used to overcome physical barriers to care during lockdowns, providing higher levels of intensive or specialised, family-centred care [45]. Actionable information systems were also described as contributing to referral efficiency between sending and receiving facilities across levels of MNH facility care [54–56]. No standardised indicators determining maternal and/or neonatal levels of care were found among the identified reports that distinguished between MNH levels of care.

Functional referral systems (quality domain three) also provide the critical linkage between maternal and newborn levels of care. Reports frequently used related descriptors at the hospital and health facility levels, including geography, referral, transfer, transport, travel time, distance, and ‘regionalisation’ (*e.g.* regional hospital or centre). A recurrent theme was the importance of strengthening referral linkages, including coupling preventive community work with improving the quality of facility care, as well as strengthening transport linkages before, during, and after facility births [57,58]. Further, we identified 51 papers (31.5%) discussing ‘referral’ and 78 papers (48.1%), ‘regionalisation.’

### Experience of care

Some descriptors were found, though fewer than in other domains, relating to levels of care that included the dimension of experience of care through respect and preservation of dignity [13,14].

Effective communication (quality domain four) was not frequently reported. One Canadian study discussed the importance of provider-family communication during transfers between levels of care [59]. Otherwise, effective communication was only discussed in terms of its absence being a bottleneck to effective MNH care [60,61].

Respect and preservation of dignity (quality domain five) in levels of MNH care was established through discussion of respectful maternity care, with included papers also citing ‘family-centred’ [45], ‘patient-centred’ [62], and ‘client-centred’ [63] care. Separation of mother and baby due to one of the two requiring a higher level of care was reported to cause feelings of frustration and disrespect [64].

Emotional support (quality domain six) emerged as a component of holistic maternal care in relation to the social and psychological impacts of pregnant women categorised as obstetric high-risk being transferred from their community to a higher level of care [65]. In relation to emotional support, one report specifically designated four levels of evidence-based psychological care for women experiencing obstetric complications based on their conditions [66].

### Additional quality processes

Competent and motivated human resources (quality domain seven) were frequent descriptors for MNH levels of care, mentioned in 72 papers (44.4%).

One report from a conflict-affected area defined the highest level of care not by place or intervention, but by the skill level of the human resources (*e.g.* maternal health workers) [67]. Other reports linked the health worker cadre with the place of care to describe the lowest level of care, predominantly in higher mortality settings: Traditional birth attendants at health huts or health posts [67,68], skilled birth attendants at home or in community health centres [43], midwives at midwife obstetric units [60,63], community health workers running community-based health planning and services zones [26–28], and ‘village health team/services’ or female community health volunteers operating in different health systems [69]. There was high variation between countries in the cadre at the lowest level of MNH care.

Essential physical resources (quality domain eight) were also frequent descriptors, for level-specific MNH facility care, found in 83 papers (51.2%). Collectively, physical resources distinguished newborn ‘special care’ from ‘neonatal intensive care units.’ Specific examples included routine ward care for low-risk patients [70] and wards equipped with extracorporeal membrane oxygenation equipment for the sickest patients [71].

### Outcomes

We found that person-centred and facility outcomes were related in various ways to levels of MNH care in the reports identified. Patient volume was commonly described in terms of outcomes (*e.g.* obstetric haemorrhage), and the levels of care hierarchy were described in terms of facility volume and output [72]. Facility-level drivers for improved outcomes included strengthening lower levels of care to reduce neonatal mortality rates [73] and increasing in-utero referral and transport between levels of care to ensure better outcomes than postpartum referral and transport [50]. Patient demographic characteristics, a salient theme in vivo, were frequently associated with MNH outcome stratification. One report from the USA identified neglecting to escalate clients of colour to risk-appropriate levels of care as a determinant of disparities in MNH outcomes [74]. Three predominant categories of characteristics were used to distinguish between hierarchical levels of maternal and neonatal care across included reports: facility characteristics (cited 157 times across 83 papers), clinical patient characteristics (cited 110 times across 71 papers), and human resource characteristics (cited 110 times across 72 papers). Volume and geography/distance were also cited in distinguishing levels, though they are not as salient [50]. Similarly, potential bottlenecks in the implementation of best emergency obstetric care practices included a lack of personnel or competence among them, inadequate essential physical resources (*e.g.* products and/or technologies), and high patient volume [43,54].

### Objective three: exploring levels of care for the maternal newborn dyad

Among 162 reports, levels of care were described in 63 (38.9%) for neonatal care, 50 (30.9%) for maternal care, and 44 (27.2%) reports for both maternal and neonatal care. The remaining five reports (3.1%) discussed concepts related but not explicit to MNH levels of care (*e.g.* regionalisation of care, networks of care, *etc.*).

Several papers highlighted the concept of the maternal-newborn dyad and how the mother and neonate move together or separately through levels of care [34,75–77]. In general, correlation between levels of care for women and newborns was advocated, whilst acknowledging the challenges of correlating variation in criteria to distinguish levels and regionalisation of care for neonatal *vs.* maternal care [78–80]. Several reports from higher-income settings state levels and regionalisation for neonatal care have received more attention than obstetric care [31,79,81,82]. The aspiration to integrate a maternal-neonatal network or system of levels of care was a stated priority to ensure optimal dyad outcomes [31,33,38,83] and to avoid fragmentation of care and resources [47]. Extra emphasis on *in utero* transfer was sometimes articulated, as was prioritisation of the woman or baby for transfer to a higher-level facility [82]. Very few reports argued against the designation of levels of care coordinated between mother and foetus [31,84] to prioritise the mother [33]. Mostly, examples were given of efforts to coordinate more effectively (*e.g.* USA has recently made efforts to incorporate maternal care into neonatal levels of care) [34,76,77].

## DISCUSSION

### Summary of evidence

In this scoping review, we summarised peer-reviewed literature regarding MNH levels of care (n = 162) published between 2009 and 2021. We found three to four levels of facility MNH care the most commonly reported, including those from settings with the lowest stillbirth, maternal, and neonatal mortality rates. In these reports, levels of care were characterised by both routine and emergency care criteria, across many domains of the WHO quality-of-care framework [13,14]. These findings can be used to strengthen the health system and accelerate progress for women and newborns towards the globally agreed goals.

Reported MNH levels of care varied in both the number reported within and between countries. These discrepancies could be due to health system reforms, parallel ministries or systems, differing definitions of levels of care, descriptions including *vs.* excluding non-facility levels of care, descriptions of emergency-specific designations (*e.g.* BEmONC/CEmONC) *vs.* MNH care, including routine and emergency services, maternal/neonatal care being described together *vs.* separately, or descriptions of recommended *vs.* actual operationalised levels of care (Table S7 in the **Online Supplementary Document**).

The lowest level of MNH care varied by context, with some contexts including non-facility levels and others excluding them. The centrality of MNH care within community healthcare was evident in the identified literature [85]. Emphasis on improving access to emergency care and institutional deliveries should not preclude recognition of the importance of community-based care. Ensuring high-quality care across the ‘household to hospital continuum of care’ is critical to achieve universal coverage by integrating primary healthcare with higher-level facility care [86].

Levels of MNH care described in these manuscripts included both routine and complication-related care. For emergency-specific designated levels of care, the two-level BEmONC and CEmONC structure was mentioned in only six reports since 2009. The EmOC framework was designed in 1997, reaffirmed in 2009, and is currently being revised [7-9], with the two levels of care determined by tracer clinical emergency intervention signal functions. The highest level, CEmOC, is achieved by performing caesarean sections and blood transfusions, in addition to the BEmONC signal functions [9]. Specifically, critical and/or intensive care has emerged as an empirical, categorical need across MNH systems to care for the sickest women and newborns [39,42]. As countries transition across the maternal newborn stillbirth transition, further reductions in preventable maternal and newborn mortality and stillbirth rates will not be possible without access to high-quality special and intensive care [87].

Approaching MNH levels of care while considering quality of care, in both experience and provision, is important for improved outcomes for women and newborns. We found that countries’ definition of their MNH levels of care was articulated with other characteristics, beyond tracer clinical emergency interventions. These characteristics aligned with the WHO quality-of-care framework, including human and physical resources [13,14]. Recognising that poor quality of care is a major contributor to preventable mortality and morbidity, measuring other criteria beyond signal functions may contribute to the improvement in quality of care needed. Multidimensional quality of care is needed at every level of the health system, and describing level-specific quality domains could be helpful for programme planning.

High-quality MNH care is safe, effective, timely, efficient, equitable and people-centred [13]. We found that integrating maternal and newborn people-centred care across MNH levels of care, emphasising the importance of avoiding separation of the mother and baby, leads to better maternal and newborn outcomes. Historically, the individual woman or newborn has been prioritised in regionalisation efforts, with neonatal care levels receiving more attention than maternal care levels [31,79,81,82]. Instead, a contextualised, networked maternal-neonatal dyad-levels-of-care approach is needed [64,78]. This aligns with the recent focus on strengthening respectful and family-centred care. Integrated MNH levels of care should be included in emerging conceptualisations of health system organisation, using a programmatic transition framework [87].

### Strengths and limitations

To our knowledge, our study is the first scoping review specific to levels of maternal and neonatal care; however, it has several potential limitations. We introduced potential language bias by restricting publications to English, Spanish, Portuguese, French, and Arabic, which could bias results toward regions where these languages are predominantly spoken. Google Scholar search results were limited to the first 200 due to the high volume. We focused on peer-reviewed publications to identify the latest evidence on levels of care thinking, but we recognise this might have excluded relevant sources and contributed to publication bias. It was beyond the scope of this study to review grey literature, including country MNH and wider health system policy documents, to understand how MNH levels of care are incorporated within the health system. We recognise that excluding grey literature may have led to the omission of valuable data, such as government reports and policy documents, which can offer insights that peer-reviewed studies alone may not.

Although the study had a global focus, the identified studies were not balanced across all geographies, with more than 50% from the Americas (and among these, many from North America), 30% from Africa, and fewer from Europe and Asia. The publications we identified spanned a 12-year period, and the country mortality rates we used to stratify may reflect more recent revisions to MNH levels of care. In line with scoping review methodology, we did not formally assess the quality of the evidence included, as is typically done in systematic reviews.

In addition, robustly addressing the role of inequity for marginalised groups and structural inequities was outside the scope of this review. The literature reviewed in this study lacked coverage of racial and ethnic disparities in MNH care, and the authors strongly feel that more research is needed on this topic as it relates to access to the levels of care available. For example, race and ethnicity are vital characteristics in the country-specific operationalisation of levels of care. Further research into equitable risk-appropriate escalation to appropriate MNH facility levels of care is paramount to the advancement of reproductive justice across maternal and neonatal outcomes.

## CONCLUSIONS

In scoping the literature on MNH levels of care, there is variation within and between countries in the number of levels described and the ways they are characterised. Among the identified countries, three or four levels of care were most often reported, including those in the lowest mortality settings. Levels of MNH care are characterised by multiple domains of the WHO quality-of-care framework, including human and physical resources, interventions, and referral structures from the community to the facility. While the dimensions of MNH care provision may differ by level, the experience of care for women and newborns needs to be prioritised at every level of care. Integration of care for the maternal-newborn dyad is a needed focus which will support family-centred care with better outcomes for all. As policymakers and practitioners plan MNH care services, these results may inform health system organisation across three or more levels of care, including intensive care at the highest level. Integration of maternal and newborn services must be a priority across all geographies to strengthen person- and family-centred care and accelerate progress for every woman and every newborn everywhere.

## Additional material


Online Supplementary Document
