# Supplementary material for: Levels of care for maternal and neonatal healthcare: a scoping review
Source: J Glob Health. 2026 Mar 20;16:04035. doi: 10.7189/jogh.16.04035 (PMC13003708; doi:10.7189/jogh.16.04035)
Supplement: Online Supplementary Document [file jogh-16-04035-s001.pdf]

Supplement to: Mackin S, Day LT, Dekker C, Gouse I, Maina M, Aluvaala J, Moran A; Revisioning Emergency Obstetric and Newborn Care (EmONC) levels-of-care workstream. Levels of care for maternal and neonatal health care: a scoping review. J Glob Health. 2026;16:04035.

Online Supplementary Material

Levels of care for maternal and neonatal health care: A scoping review

Shaina Mackin*, Louise Tina Day*, Caity Dekker, Isabel Gouse, Michuki Maina, Jalemba Aluvaala±, Allisyn Moran±, Revisioning Emergency Obstetric and Newborn Care (EmONC) levels-of-care workstream

*Joint first
±Joint last

Checklist

Checklist S1: Preferred Reporting Items for Systematic reviews and Meta-Analyses extension for Scoping Reviews (PRISMA-ScR) Checklist, for levels of care for maternal and neonatal health care: A scoping review 2

Figures and Tables

Figure S1: Number of MNH Facility Levels of Care Identified by Region 3
Table S1: Composition of the Revisioning EmONC Levels of Care Working Group 4
Table S2: Search Strings for levels of care for maternal and neonatal health care: A scoping review 7
Table S3: Data Extraction Tool for levels of care for maternal and neonatal health care: A scoping review 8
Table S4: Reports included (n=162) in levels of care for maternal and neonatal health care: A scoping review 9
Table S5: Number of reports identified by Maternal and Newborn Health levels of care reported, categorized by region (n=162 reports) 17
Table S6: Number of reports identified by Maternal and Newborn Health facility levels of care reported, categorized by mortality outcomes (n=145 countries discussed with specified number of levels across 162 reports) 18
Table S7: Number of MNH levels (including non-facility) operationalized & recommended across reports 19

Checklist S1: Preferred Reporting Items for Systematic reviews and Meta-Analyses extension for Scoping Reviews (PRISMA-ScR) Checklist, for levels of care for maternal and neonatal health care: A scoping review

Preferred Reporting Items for Systematic reviews and Meta-Analyses extension for Scoping Reviews (PRISMA-ScR) Checklist

SECTION	ITEM	PRISMA-ScR CHECKLIST ITEM	REPORTED ON PAGE #
**TITLE**			
Title	1	Identify the report as a scoping review.	1
**ABSTRACT**			
Structured summary	2	Provide a structured summary that includes (as applicable): background, objectives, eligibility criteria, sources of evidence, charting methods, results, and conclusions that relate to the review questions and objectives.	2
**INTRODUCTION**			
Rationale	3	Describe the rationale for the review in the context of what is already known. Explain why the review questions/objectives lend themselves to a scoping review approach.	3-4
Objectives	4	Provide an explicit statement of the questions and objectives being addressed with reference to their key elements (e.g., population or participants, concepts, and context) or other relevant key elements used to conceptualize the review questions and/or objectives.	5
**METHODS**			
Protocol and registration	5	Indicate whether a review protocol exists; state if and where it can be accessed (e.g., a Web address); and if available, provide registration information, including the registration number.	Not registered
Eligibility criteria	6	Specify characteristics of the sources of evidence used as eligibility criteria (e.g., years considered, language, and publication status), and provide a rationale.	6
Information sources*	7	Describe all information sources in the search (e.g., databases with dates of coverage and contact with authors to identify additional sources), as well as the date the most recent search was executed.	6
Search	8	Present the full electronic search strategy for at least 1 database, including any limits used, such that it could be repeated.	6
Selection of sources of evidence†	9	State the process for selecting sources of evidence (i.e., screening and eligibility) included in the scoping review.	6
Data charting process‡	10	Describe the methods of charting data from the included sources of evidence (e.g., calibrated forms or forms that have been tested by the team before their use, and whether data charting was done independently or in duplicate) and any processes for obtaining and confirming data from investigators.	6
Data items	11	List and define all variables for which data were sought and any assumptions and simplifications made.	6
Critical appraisal of individual sources of evidence§	12	If done, provide a rationale for conducting a critical appraisal of included sources of evidence; describe the methods used and how this information was used in any data synthesis (if appropriate).	7
Synthesis of results	13	Describe the methods of handling and summarizing the data that were charted.	7

Figure S1: Number of MNH Facility Levels of Care Identified by Region

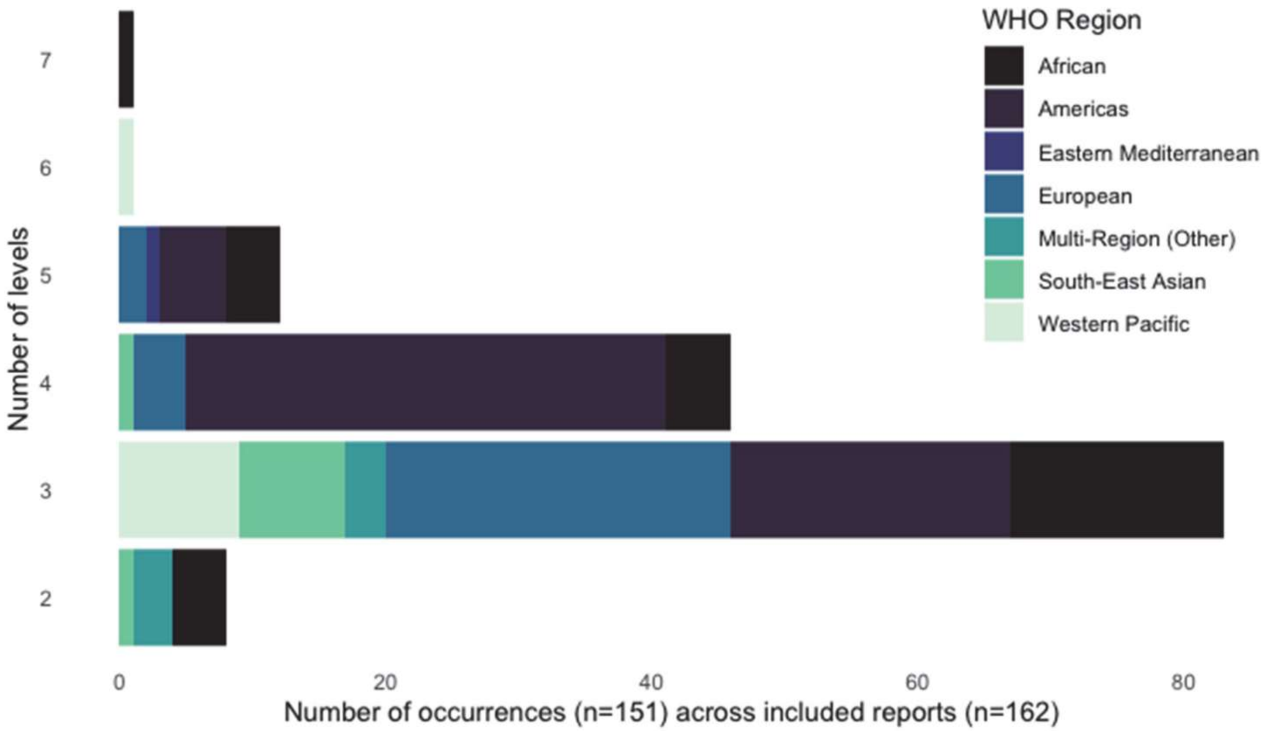

Note: "Multi-Region" was defined as 'global', 'low-resource', or 'LMIC' settings; occurrences without specified numbers of levels were dropped.

Table S2: Composition of the Revisioning EmONC Levels of Care Working Group

Name	Credentials	Organization	Job Title	Training, area of expertise	Role
**Allisyn Moran (AM)**	PhD	World Health Organization, Geneva, Switzerland	Unit Head, Maternal Health	Public health, maternal health	Co-chair
**Jalemba Aluvaala (JA)**	DPhil	University of Nairobi, KEMRI-Wellcome Trust Research Programme, Kenya	Senior Lecturer	Paediatrics, Epidemiology, Health Services Research	Co-chair
**Louise Tina Day (LTD)**	PhD	London School of Hygiene & Tropical Medicine (LSHTM), UK	Assistant Professor	Obstetrics, Paediatrics, Epidemiology. Quality of care/ data	Member
**Shaina Mackin (SM)**	MPH	Averting Maternal Death and Disability (AMDD) Program, Mailman School of Public Health, Columbia University, USA	Research Assistant	Public Health, Comparative Effectiveness and Outcomes Research	Member
**Caity Dekker (CD)**	MPH	Averting Maternal Death and Disability (AMDD) Program, Mailman School of Public Health, Columbia University, USA	Research Assistant	Public Health	Member
**Michuki Maina (MM)**	PhD	KEMRI Wellcome Trust Programme, Kenya	Clinical Research Fellow	Paediatrics, Neonates, Public Health, Health Systems	Member
**Isabel Gouse (IG)**	MPH	Averting Maternal Death and Disability (AMDD) Program, Mailman School of Public Health, Columbia University, USA	Research Assistant	Public Health	Member
Patience Afulani (PA)	PhD	University of California, San Francisco, USA	Associate Professor	Public Health	Member
Kaosar Afsana (KA)*	PhD	BRAC University, Bangladesh	Professor	Public Health	Member
Patsy Bailey (PB)	PhD	Averting Maternal Death and Disability (AMDD) Program, Mailman School of Public Health, Columbia University, USA	Consultant	Epidemiologist	Member
Lynn Freedman (LF)	PhD	Averting Maternal Death and Disability (AMDD) Program, Mailman School of	Professor	Health and human rights Maternal health, Sexual and reproductive health and rights	Member

Name	Credentials	Organization	Job Title	Training, area of expertise	Role
		Public Health, Columbia University, USA			
Tedbabe Hailegebriel (TH)	MPH	UNICEF Headquarters, New York, USA	Unit Head	Paediatrics, Public Health	Member
Elimase Kamanga (EK)	Master's degree	White Ribbon Alliance, Malawi	National Lead	Public Health	Member
Mary Kinney (MK)	PhD	University of the Western Cape, School of Public Health, South Africa	Research consultant	Health policy and systems, quality of care	Member
Ornella Lincetto (OL)	MPH	Independent Consultant, Italy	Consultant	Paediatrics, Public Health	Member
Samantha Lobis (SL)	MPH	Averting Maternal Death and Disability (AMDD) Program, Mailman School of Public Health, Columbia University, USA	Consultant	Public health; maternal and reproductive health; program design; monitoring and evaluation; health facility assessments	Member
Zoe Matthews (ZM)	PhD	Global Health and Social Statistics, University of Southampton, UK	Professor	Statistics, Public Health	Member
Jean-Pierre Monet (JPM)	MPH	UNFPA, New York	Maternal Health	Public health, maternal health	
Isabelle Moreira (IM)	MPH	UNFPA, Senegal	Consultant		Member
Sarah Moxon (SM)	PhD	London School of Hygiene & Tropical Medicine (LSHTM), UK	Assistant Professor	Neonatal nursing, public health, health systems	Member
Kristine Nilsen (KN) *	MPH	Averting Maternal Death and Disability (AMDD) Program, Mailman School of Public Health, Columbia University, USA	Consultant	Public Health	Member
Cynthia Boschi Pinto (CBP) *	DS	Averting Maternal Death and Disability (AMDD) Program, Mailman School of Public Health, Columbia University, USA	Consultant	Public Health	Member
Kate Ramsey (KR)	PhD	Averting Maternal Death and Disability (AMDD) Program, Mailman School of Public Health, Columbia University, USA	Consultant	Public health; maternal and reproductive health; program design; monitoring and	Member

Name	Credentials	Organization	Job Title	Training, area of expertise	Role
				evaluation; health facility assessments	
Jennifer Requejo (JR)	PhD	Johns Hopkins University, USA	Senior Scientist	Epidemiology, Public Health	Member
Sanam Roder-DeWan (SRD)	PhD	Dartmouth University, USA	Associate Professor	Family Practice, Health Systems	Member
Suzanne Stalls (SS)	MA	Jhpiego, USA	Head Maternal Health	Midwifery, Public Health	Member
Caitlin Warthin (CW)	MPH	Averting Maternal Death and Disability (AMDD) Program, Mailman School of Public Health, Columbia University, USA	Deputy Director	Public Health	Member

*Acknowledgements

Table S3: Search Strings for levels of care for maternal and neonatal health care: A scoping review

Database	Search terms
**Embase**	(((maternal OR newborn) AND ("levels of care" OR "networks of care" OR ("service delivery" AND models))) OR ("regionalization" and health)) AND [2009-2021]/py
	("integrated networks of care") OR ("maternal and newborn health services") AND [2020-2021]/py
**Medline**	(((maternal OR newborn) AND ("levels of care" OR "networks of care" OR ("service delivery" AND models))) OR ("regionalization" and health)) AND (("2009"[Date-Publication]: "3000"[Date-Publication]))
	(("integrated networks of care") OR ("maternal and newborn health services")) AND (("2020/06"[Date-Publication]: "3000"[Date-Publication]))
**Google Scholar**	(((maternal OR newborn) AND ("levels of care" OR "networks of care" OR ("service delivery" AND models))) OR ("regionalization" and health)) (Range: 2009-2021) (Sorted by Relevance)
	("integrated networks of care") OR ("maternal and newborn health services") (Range: 2020-2021) (Sorted by Relevance)
**Cochrane**	(((maternal OR newborn) AND ("levels of care" OR "networks of care" OR ("service delivery" AND models))) OR ("regionalization" and health)) AND 2009-2021
	("integrated networks of care") OR ("maternal and newborn health services") AND 2020-2021

Table S4: Data Extraction Tool for levels of care for maternal and neonatal health care: A scoping review

Author(s)	
Title	
Year of publication	
Search strategy (e.g., database, expert recommendation)	
Type of literature (e.g., peer-reviewed, gray literature)	
Source (e.g., journal name)	
Study design	
Geographic location	
Unit(s) of analysis	
Client focus (e.g., maternal, neonatal, both)	
Scoping review objectives met (I, II, III, and/or IV)	
Number of MNH levels of care	
Definitions and conceptualizations of MNH levels of care	
Details on the maternal-newborn dyad	
Measures identified to determine MNH levels of care	
COVID-19 mentioned (Y/N)	
Priority (low, medium, high)	

Table S5: Reports included (n=162) in levels of care for maternal and neonatal health care: A scoping review

Author(s)	Title	Year	Source
Aboudi et al.	Impact of neonatologist availability on preterm survival without morbidities	2018	Journal of Perinatology
ACOG	ACOG committee opinion no. 726: hospital disaster preparedness for obstetricians and facilities providing maternity care	2017	Obstetrics & Gynecology
Ameyaw et al.	Quality and women's satisfaction with maternal referral practices in sub-Saharan African low and lower-middle income countries: a systematic review	2020	BMC Pregnancy and Childbirth
Amoro et al.	Bypassing primary healthcare facilities for maternal healthcare in North West Ghana: socio-economic correlates and financial implications	2021	BMC Health Services Research
Anto-Ocrah et al.	A woman's worth: an access framework for integrating emergency medicine with maternal health to reduce the burden of maternal mortality in sub-Saharan Africa	2020	BMC Emergency Medicine
Arbio et al.	Profile of spontaneous demand for services among infants younger than 30 days old at a children's tertiary care hospital	2017	Archivos Argentinos de Pediatria
Azzuqa et al.	Use of telemedicine for subspecialty support in the NICU setting	2021	Seminars in Perinatology
Badgery-Parker et al.	Patterns and outcomes of preterm hospital admissions during pregnancy in NSW, 2001-2008	2012	Medical Journal of Australia
Baird, S.; Martin, S.	Framework for critical care in obstetrics	2018	Journal of Perinatal & Neonatal Nursing
Bajaña et al.	Recommendations on organization, design, characteristics and operation of neonatology services	2021	Andes Pediatrica
Banke-Thomas et al.	Influence of travel time and distance to the hospital of care on stillbirths: a retrospective facility-based cross-sectional study in Lagos, Nigeria	2021	BMJ Global Health
Barfield et al.	Levels of neonatal care	2012	Pediatrics
Barnea et al.	From fragmented levels of care to integrated health care: framework toward improved maternal and newborn health	2021	International Journal of Gynecology & Obstetrics
Bellini & Ramenghi	The neonatal transport index could be used as a reference tool for the Italian perinatal care regionalisation plan	2018	Acta Paediatrica
Bezner et al.	Pediatric surgeons' attitudes toward regionalization of neonatal surgical care	2014	Journal of Pediatric Surgery
Bhakoo & Kumar	Current challenges and future prospects of neonatal care in India	2013	Indian Journal of Pediatrics
Bhatta et al.	The logarithmic spiral of networks of care for expectant families in rural Nepal: a descriptive case study	2020	Health Systems & Reform
Bizzarro & Gallagher	Why so little progress in regionalization of perinatal care when transport of high-risk neonates remains a substantial risk?	2020	Journal of Perinatology

Author(s)	Title	Year	Source
Blackmon et al.	Hospital neonatal services in the United States: variation in definitions, criteria, and regulatory status, 2008	2009	Journal of Perinatology
Blondel et al.	Organisation of obstetric services for very preterm births in Europe: results from the MOSAIC project	2009	BJOG: An International Journal of Obstetrics and Gyanecology
Bolbocean et al.	Perinatal regionalization and implications for long-term health outcomes in cerebral palsy	2016	Canadian Journal of Neurological Sciences
Bourque et al.	Prevalence and predictors of back-transport closer to maternal residence after acute neonatal care in a regional NICU	2019	Maternal and Child Health Journal
Bourque & Hwang	Underuse versus overuse of neonatal intensive care: what is the right amount?	2018	The Journal of Pediatrics
Brantley et al.	Perinatal regionalization: a geospatial view of perinatal critical care, United States, 2010-2013	2017	American Journal of Obstetrics & Gynecology
Bronstein et al.	Improving perinatal regionalization for preterm deliveries in a Medicaid covered population: initial impact of the Arkansas ANGELS intervention	2011	Health Services Research
Bronstein et al.	Improving perinatal regionalization: 10 years of experience with an Arkansas initiative	2020	Journal of Perinatology
Busumani & Mundagowa	Outcomes of pregnancy-related referrals from rural health facilities to two central hospitals in Harare, Zimbabwe: a prospective descriptive study	2021	BMC Health Services Research
Carlisle & Rowell	Ethical challenges in regionalization of pediatric surgical care	2019	Current Opinion in Pediatrics
Catalano et al.	Implementing CDC's Level of Care Assessment Tool (LOCATe): a national collaboration to improve maternal and child health	2017	Journal of Women's Health
Cattaneo et al.	Report on an international workshop on kangaroo mother care: lessons learned and a vision for the future	2018	BMC Pregnancy and Childbirth
Cavallaro et al.	What the percentage of births in facilities does not measure: readiness for emergency obstetric care and referral in Senegal	2020	BMJ Global Health
Chang	Regionalization of neonatal intensive care in Korea	2011	Korean Journal of Pediatrics
Chilvers	Planning framework for human resources for health for maternal and newborn care	2014	LSHTM Research Online
Clapp et al.	The effect of hospital acuity on severe maternal morbidity in high-risk patients	2018	American Journal of Obstetrics & Gynecology
Clapp et al.	Comparison of maternal and neonatal subspecialty care provision by hospital	2021	American Journal of Perinatology
Coutts et al.	What is stopping us? An implementation science study of kangaroo care in British Columbia's neonatal intensive care units	2021	BMC Pregnancy and Childbirth
Craig & Smith	Risk-adjusted/neuroprotective care services in the NICU: the elemental role of the neonatal therapist (OT, PT, SLP)	2020	Journal of Perinatology
Daniels & Abuosi	Improving emergency obstetric referral systems in low and middle income countries: a qualitative study in a tertiary health facility in Ghana	2020	BMC Health Services Research
Daniels et al.	Steps toward a national disaster plan for obstetrics	2014	Obstetric Anesthesia Digest

Author(s)	Title	Year	Source
Deindl & Diemert	From structural modalities in perinatal medicine to the frequency of preterm birth	2020	Seminars in Immunopathology
Diamond-Smith et al.	Clinical and perceived quality of care for maternal, neonatal and antenatal care in Kenya and Namibia: the service provision assessment	2016	Reproductive Health
Dillip et al.	Can formalizing links among community health workers, accredited drug dispensing outlet dispensers, and health facility staff increase their collaboration to improve prompt access to maternal and child care? a qualitative study in Tanzania	2017	BMC Health Services Research
Dosani et al.	Parental perception of neonatal transfers from level 3 to level 2 neonatal intensive care units in Calgary, Alberta: qualitative findings	2021	BMC Health Services Research
Easter et al.	Provider attitudes on regionalization of maternity care: a national survey	2021	Maternal and Child Health Journal
Evans et al.	Quantifying the potential bias when directly comparing standardised mortality ratios for in-unit neonatal mortality	2013	PLOS One
Fasawe et al.	Applying a client-centered approach to maternal and neonatal networks of care: case studies from urban and rural nigeria	2020	Health Systems & Reform
Fawcus	Practical approaches to managing postpartum haemorrhage with limited resources	2019	Best Practice & Research Clinical Obstetrics and Gynaecology
Fawcus et al.	Trends in maternal deaths from obstetric haemorrhage in South Africa 2008-2010	2012	Obstetrics & Gynaecology Forum
Gomez Lopez	Psychological care guide for women with obstetric complications	2018	Perinatologia y Reproducion Humana
Gage et al.	In low- and middle-income countries, is delivery in high-quality obstetric facilities geographically feasible?	2019	Health Affairs
Gente et al.	Nationwide survey of neonatal transportation practices in Italy	2019	Italian Journal of Pediatrics
Gething et al.	Geographical access to care at birth in Ghana: a barrier to safe motherhood	2012	BMC Public Health
Goldenberg et al.	Reducing maternal mortality from preeclampsia and eclampsia in low-resource countries--what should work?	2015	Acta Obstetricia et Gynecologica Scandinavica
Gould	The role of regional collaboratives: the California Perinatal Quality Care Collaborative model	2010	Clinical Perinatology
Gouyon et al.	Neonatal problems of late and moderate preterm infants	2012	Seminars in Fetal & Neonatal Medicine
Grytten et al.	Regionalization and local hospital closure in Norwegian maternity care: the effect on neonatal and Infant mortality	2014	Health Services Research
Hall-Barrow et al.	Telemedicine and neonatal regionalization of care - ensuring that the right baby gets to the right nursery	2009	Pediatric Annals
Hall et al.	Neonatal regionalization through telemedicine using a community based research and education core facility	2010	Ethnicity & Disease
Handley et al.	Identifying individual hospital levels of maternal care using administrative data	2021	BMC Health Services Research
Handley et al.	Regionalization of care and the maternal-infant dyad disconnect	2019	JAMA

Author(s)	Title	Year	Source
Hankins et al.	Maternal mortality, near misses, and severe morbidity: lowering rates through designated levels of maternity care	2012	Obstetrics & Gynecology
Hanson & Schellenberg	Redesigning maternal health services: is centralisation the answer in low-resource settings?	2019	BMJ Global Health
Harrison et al.	Regional variation in neonatal intensive care admissions and the relationship to bed supply	2018	The Journal of Pediatrics
Helenius et al.	Amount of antenatal care days in a context of effective regionalization of very preterm deliveries	2016	Journal of Pediatrics
Holmstrom & Phibbs	Regionalization and mortality in neonatal intensive care	2009	Pediatric Clinics of North America
Hossain et al.	Outcome comparison of very preterm infants cared for in the neonatal intensive care units in Australia and New Zealand and in Canada	2015	Journal of Paediatrics and Child Health
Ijadunola et al.	New paradigm old thinking: the case for emergency obstetric care in the prevention of maternal mortality in Nigeria	2010	BMC Women's Health
Isayama	The clinical management and outcomes of extremely preterm infants in Japan: past, present, and future	2019	Translational Pediatrics
James et al.	Identifying women requiring maternity high dependency care	2011	Midwifery
Jantsch et al.	Health care network (dis)articulation in late and moderate prematurity	2021	Revista Brasileira de Enfermagem
Jha et al.	A study on maternal and perinatal outcome in referred obstetric cases of gestational age more than 28 weeks in a rural medical college hospital	2018	Journal of South Asian Federation of Obstetrics and Gynecology
Kaiser et al.	The effects of maternity waiting homes on the health workforce and maternal health service delivery in rural Zambia: a qualitative analysis	2019	Human Resources for Health
Kane et al.	Making effective referrals happen: a theory-informed policy analysis	2020	Health Policy and Planning
Kastenberg et al.	Effect of deregionalized care on mortality in very low-birth-weight infants with necrotizing enterocolitis	2015	JAMA Pediatrics
Kc et al.	State of maternal, newborn and child health programmes in Nepal: what may a continuum of care model mean for more effective and efficient service delivery?	2011	Journal of Nepal Health Research Council
Keene et al.	Developing recommendations for neonatal inpatient care service categories: reflections from the research, policy and practice interface in Kenya	2019	BMJ Global Health
Kern-Goldberger et al.	Racial disparities in maternal critical care: are there racial differences in level of care?	2021	Journal of Racial and Ethnic Health Disparities
Kern-Goldberger et al.	An assessment of baseline risk factors for peripartum maternal critical care interventions	2020	The Journal of Maternal-Fetal & Neonatal Medicine
Kilpatrick et al.	Levels of maternal care: obstetric care consensus no. 9	2019	Obstetrics & Gynecology
Kim et al.	Regionalization of pediatric emergency care in Korea	2011	Korean Journal of Pediatrics
Kim et al.	Telemedicine collaboration improves perinatal regionalization and lowers statewide infant mortality	2013	Journal of Perinatology
Kornelsen et al.	Does distance matter? increased induction rates for rural women who have to travel for intrapartum care	2009	Journal of Obstetrics & Gynecology Canada

Author(s)	Title	Year	Source
Korst et al.	Cross-sectional survey of California childbirth hospitals: implications for defining maternal levels of risk-appropriate care	2015	American Journal of Obstetrics & Gynecology
Kozhimannil et al.	Location of childbirth for rural women: implications for maternal levels of care	2016	American Journal of Obstetrics & Gynecology
Kroelinger et al.	Comparison of state risk-appropriate neonatal care policies with the 2012 AAP policy statement	2018	Journal of Perinatology
Kroelinger et al.	Designation of neonatal levels of care: a review of state regulatory and monitoring policies	2020	Journal of Perinatology
Kroelinger et al.	Seven years later: state neonatal risk-appropriate care policy consistency with the 2012 American Academy of Pediatrics Policy	2021	Journal of Perinatology
Kunz et al.	The changing landscape of perinatal regionalization	2020	Seminars in Perinatology
Kunz et al.	Predicting successful neonatal retro-transfer to a lower level of care	2019	The Journal of Pediatrics
Kunz et al.	Quantifying the variation in neonatal transport referral patterns using network analysis	2021	Journal of Perinatology
Kunz et al.	Network analysis: a novel method for mapping neonatal acute transport patterns in California	2017	Journal of Perinatology
LaGamma et al.	Making a business case for a model of regionalization with neonatologist availability at all facilities: one institution's financial tell-all	2018	Journal of Perinatology
Lasswell et al.	Perinatal regionalization for very low-birth-weight and very preterm infants: a meta-analysis	2010	JAMA
Lazariu et al.	Severe maternal morbidity: a population-based study of an expanded measure and associated factors	2017	PLOS One
Leone & Abreu	The term newborn: delivery room triage and transitions of care	2021	Clinics in Perinatology
Little	Variation, perinatal regionalization and total cohort accountability	2009	Journal of Perinatology
Long et al.	Maternity care system in Maputo, Mozambique: plans and practice?	2017	Cogent Medicine
Lorch	Perinatal legislative policies and health outcomes	2017	Seminars in Perinatology
M'Rithaa et al.	Development spots in communication during the management of the intrapartum period: an interpretive multiple case study in a developing context	2017	African Journal of Primary Health Care & Family Medicine
Marcus & Clow	Response times of ambulances to calls from Midwife Obstetric Units of the Peninsula Maternal and Neonatal Service (PMNS) in Cape Town	2009	Curationis
Martinelli et al.	Transport as a system: reorganization of perinatal assistance in Northern Lombardy	2011	The Journal of Maternal-Fetal & Neonatal Medicine
Martinez Vergara et al.	Building trust to save lives in a metro Manila public-private network of care: a descriptive case study of Quirino Recognized Partners in Quezon City, Philippines	2020	Health Systems & Reform
Mathison et al.	Variations in interfacility transport: approach to call intake, team composition, and mode of transport	2013	Clinical Pediatric Emergency Medicine
McCoy et al.	Establishing level II neonatal services in southwestern oklahoma	2014	The Journal of Oklahoma State Medical Association

Author(s)	Title	Year	Source
Menard et al.	Levels of maternal care	2015	American Journal of Obstetrics & Gynecology
Mesquita et al.	The Spanish triage system in the evaluation of neonates in paediatric emergency departments	2017	Revista Chilena de Pediatria
Moiseienko et al.	Development of perinatal medicine in Ukraine in the context of international approaches	2021	Wiadomości Lekarskie
Moxon et al.	Categorising interventions to levels of inpatient care for small and sick newborns: findings from a global survey	2019	PLOS One
Mukuru et al.	Challenges in implementing emergency obstetric care (EmOC) policies: perspectives and behaviours of frontline health workers in Uganda	2021	Health Policy and Planning
Narli et al.	Turkish Neonatal Society guideline on the safe transport of newborn	2018	Turkish Archives of Pediatrics
Neogi et al.	Does facility-based newborn care improve neonatal outcomes? a review of evidence	2012	Indian Pediatrics
Nevacinovic et al..	Impact of perinatal health care organization on mortality of low birth weight infants in the federation of bosnia and herzegovina	2011	Acta Medica Saliniana
Nnebue et al.	Availability and continuity of care for maternal health services in the primary health centres in Nnewi, Nigeria (January - March 2010)	2016	International Journal of Preventative Medicine
Nowakowski et al.	Assessment of state measures of risk-appropriate care for very low birth weight infants and recommendations for enhancing regionalized state systems	2012	Maternal and Child Health Journal
O'Malley et al.	Maternal near miss: what lies beneath?	2016	European Journal of Obstetrics & Gynecology and Reproductive Biology
Okoroh et al.	United States and territory policies supporting maternal and neonatal transfer: review of transport and reimbursement	2016	Journal of Perinatology
Okoroh et al.	US and territory telemedicine policies: identifying gaps in perinatal care	2016	American Journal of Obstetrics and Gynecology
Pandian et al.	Increased utilization of primary health care centers for birthing care in Tamil Nadu, India: a visible impact of policies, initiatives, and innovations	2013	Journal of Family Medicine and Primary Care
Perry	Fifty years of progress in neonatal and maternal transport for specialty care	2021	Journal of Obstetric, Gynecologic & Neonatal Nursing
Phibbs	Managed clinical networks in neonatal care: reduces morbidity after preterm births but regionalisation of obstetric care is key	2012	British Medical Journal
Pinto et al.	Bridging the gaps in obstetric care: perspectives of service delivery providers on challenges and core components of care inrRural Georgia	2016	Maternal Child Health Journal
Pinto et al.	Regionalization of Brazilian pediatric cardiovascular surgery	2013	Brazilian Journal of Cardiovascular Surgery
Pires et al.	Engaging the community in transforming maternal care	2021	University of Saskatchewan
Pollock et al.	Maternal severity of illness across levels of care: a prospective, cross-sectional study	2011	Australian College of Critical Care Nurses
Prathiba et al.	Referral chain of patients with obstetric emergency from primary care to tertiary care: a gap analysis	2020	Journal of Family Medicine and Primary Care

Author(s)	Title	Year	Source
Rahman et al.	Effectiveness of an integrated approach to reduce perinatal mortality: recent experiences from Matlab, Bangladesh	2011	BMC Public Health
Rashidian et al.	The effectiveness of regionalization of perinatal care services - a systematic review	2014	Public Health
Rigoli et al.	Tracking pregnant women displacements in Sao Paulo, Brazil: a complex systems approach to regionalization through the emergence of patterns	2019	BMC Medicine
Rite Gracia et al.	Niveles asistenciales y recomendaciones de minimos para la atencion neonatal	2013	Anales de Pediatria
Roder-DeWan	Decentralization and regionalization: redesigning health systems for high quality maternity care: comment on decentralization and regionalization of surgical care: a review of evidence for the optimal distribution of surgical services in low- and middle-income countries	2021	International Journal of Health Policy and Management
Rosenbloom et al.	Addressing medically underserved populations through maternal-fetal transport: a geographic analysis	2020	The Journal of Maternal-Fetal & Neonatal Medicine
Salazar et al.	Regionalization of pediatric surgery: trends already underway	2016	Annals of Surgery
Sami et al.	Understanding health systems to improve community and facility level newborn care among displaced populations in South Sudan: a mixed methods case study	2018	BMC Pregnancy and Childbirth
Schmitz et al.	Referral transit time between sending and first-line receiving health facilities: a geographical analysis in Tanzania	2019	BMJ Global Health
Scott	Obstetric transport	2016	Obstetrics & Gynecology Clinics of North America
Seacrist et al.	Quality improvement opportunities identified through case review of pregnancy-related deaths from obstetric hemorrhage	2019	Journal of Obstetric, Gynecologic, & Neonatal Nursing
Seaton et al.	Modelling neonatal care pathways for babies born preterm: an application of multistate modelling	2016	PLOS One
Sequeira D'Mello et al.	Averting maternal death and disability in an urban network of care in Dar es Salaam, Tanzania: a descriptive case study	2020	Heath Systems & Reform
Serfaty et al.	From knowledge to planning considerations: a matrix to assess health needs for the perinatal network in eastern Paris	2010	European Journal of Public Health
Sharma et al.	Quality care during labour and birth: a multi-country analysis of health system bottlenecks and potential solutions	2015	BMC Pregnancy & Childbirth
Shima et al.	Interfacility neonatal transport for convalescent care: improving regionalized care	2020	Journal of Nippon Medical School
Simioni et al.	Regionalization of perinatal health care in the province of Santa Fe, Argentina	2017	Pan American Journal of Public Health
Soliman et al.	Neonatal transport services, a cross-sectional study	2021	The Journal of Maternal-Fetal & Neonatal Medicine
Srinivas et al.	Do hospitals with a higher level of maternal care designation have better maternal outcomes?	2018	American Journal of Perinatology
Staebler	Regionalized systems of perinatal care: health policy considerations	2011	Advances in Neonatal Care

Author(s)	Title	Year	Source
Stewart et al.	Optimizing outcomes in regionalized perinatal care: integrating maternal and neonatal emergency referral, triage, and transport	2017	Current Treatment Options in Pediatrics
Suehara	Cross-regional perinatal healthcare systems: efforts in Greater Kansai Area, Japan	2010	Japan Medical Association Journal
Tappis et al.	Context matters: successes and challenges of intrapartum care scale-up in four districts of Afghanistan	2015	Global Public Health
Teela et al.	Community-based delivery of maternal care in conflict-affected areas of eastern Burma: perspectives from lay maternal health workers	2009	Social Science & Medicine
Tette et al.	The pattern of neonatal admissions and mortality at a regional and district hospital in the Upper West Region of Ghana; a cross-sectional study	2020	PLOS One
Tomé et al.	Neonatal morbi-mortality in very low birth weight in Europe: the Portuguese experience	2009	Journal of Maternal-Fetal & Neonatal Medicine
Uslu et al.	The effects of improvement efforts on regional neonatal transport conditions in Diyarbakir	2011	Turkish Archives of Pediatrics
Uzondu et al.	Female health workers at the doorstep: a pilot of community-based maternal, newborn, and child health service delivery in Northern Nigeria	2015	Global Health: Science and Practice
Van Otterloo & Connelly	Risk-appropriate care to improve practice and birth outcomes	2018	Journal of Obstetric, Gynecologic, & Neonatal Nursing
Vendittelli et al.	Policies for management of postpartum haemorrhage: the HERA cross-sectional study in France	2016	European Journal of Obstetrics & Gynecology and Reproductive Biology
Vladutiu et al.	Levels of maternal care in the United States: an assessment of publicly available state guidelines	2020	Journal of Women's Health
Wenstrom et al.	Maternal-fetal medicine workforce survey: are we ready for regionalized levels of maternal care?	2018	American Journal of Perinatology
Whitfield & Chessex	The need for public involvement when operating a regionalized neonatal care system at maximum capacity	2010	Pediatrics and Child Health
Wimmer	Levels of care for perinatal health	2020	North Carolina Medical Journal
Wright et al.	Regionalization of care for obstetric hemorrhage and its effect on maternal mortality	2010	Obstetrics & Gynecology
Zahn et al.	Levels of maternal care verification pilot: translating guidance into practice	2018	Obstetrics & Gynecology
Zaidi et al.	Public private partnerships for improving maternal and neonatal health service delivery: a review of the evidence	2013	Research and Advocacy Fund, British Council Islamabad

Table S6: Number of reports identified by Maternal and Newborn Health levels of care reported, categorized by region (n=162 reports)

A: Facility levels of MNH care only

	**Americas**		**African**		**European**		**South-east Asian**		**Western Pacific**		**Eastern Mediterr-anean**		**Multi-Region**		**Total**
	n	%	n	%	n	%	n	%	n	%	n	%	n	%	n
Unspecified	20	(24.39)	17	(36.17)	1	(3.03)	6	(37.50)	5	(33.33)	2	(66.67)	4	(40.00)	55
7 levels	0	-	1*	(2.13)	0	-	0	-	0	-	0	-	0	-	1
6 levels	0	-	0	-	0	-	0	-	1**	(6.67)	0	-	0	-	1
5 levels	5	(6.10)	4	(8.51)	2	(6.06)	0	-	0	-	1	(33.33)	0	-	12
4 levels	36	(43.90)	5	(10.64)	4	(12.12)	1	(6.25)	0	-	0	-	0	-	46
3 levels	21	(25.61)	16	(34.04)	26	(78.79)	8	(50.00)	9	(60.00)	0	-	3	(30.00)	83
2 levels	0	-	4	(8.51)	0	-	1	(6.25)	0	-	0	-	3	(30.00)	8
Total	82		47		33		16		15		3		10		206

B: All levels of MNH care (home, community, health facility)

Unspecified	20	(24.39)	18	(33.33)	1	(2.86)	6	30.00	5	(33.33)	2	(50.00)	4	(30.77)	56
7 levels	0	-	1	(1.85)	0	-	0	-	0	-	0	-	0	-	1
6 levels	0	-	2	(3.70)	0	-	1	5.00	1	(6.67)	1	(25.00)	0	-	5
5 levels	5	(6.10)	4	(7.41)	3	(8.57)	2	10.00	0	-	1	(25.00)	0	-	15
4 levels	36	(43.90)	8	(14.81)	5	(14.29)	1	5.00	0	-	0	-	1	(7.69)	51
3 levels	21	(25.61)	17	(31.48)	26	(74.29)	9	45.00	9	(60.00)	0	-	5	(38.46)	87
2 levels	0	-	4	(7.41)	0	-	1	5.00	0	-	0	-	3	(23.08)	8
Total	82		54		35		20		15		4		13		223

"Multi Region": global, low-resource, or LMIC settings
"Unspecified": discusses concepts related to levels of care without specifying number
*Uganda (Mukuru et al, 2021)
**Australia (Hankins et al, 2012)

Table S7: Number of reports identified by Maternal and Newborn Health facility levels of care reported, categorized by mortality outcomes (n=145 countries discussed with specified number of levels across 162 reports)

A: Stillbirth rates (per 1,000 total births) by levels of MNH care

	>25		>20 to 25		>12 to 20		>5 to 12		>2.5 to 5		<= 2.5	
	n	%	n	%	n	%	n	%	n	%	n	%
7 levels	0	-	0	-	1	(4.3)	0	-	0	-	0	-
6 levels	0	-	0	-	0	-	0	-	0	-	1	(5.3)
5 levels	1	(33.3)	1	(6.7)	3	(13.0)	0	-	5	(6.2)	2	(10.5)
4 levels	0	-	2	(13.3)	4	(17.4)	0	-	40	(49.4)	0	-
3 levels	2	(66.7)	9	(60.0)	13	(56.5)	4	(100.0)	36	(44.4)	16	(84.2)
2 levels	0	-	3	(20.0)	2	(8.7)	0	-	0	-	0	-
Total	3		15		23		4		81		19	

B: Obstetric transition stages (maternal mortality rates per 100,000 live births) by levels of MNH care

	Stage I (>999)		Stage II (300-999)		Stage III (50-299)		Stage IV (5-49)		Stage V (<5)	
7 levels	0	-	1	(4.3)	0	-	0	-	0	-
6 levels	0	-	0	-	0	-	1	(1.1)	0	-
5 levels	0	-	4	(17.4)	1	(5.6)	5	(5.3)	2	(22.2)
4 levels	0	-	3	(13.0)	3	(16.7)	40	(42.6)	0	-
3 levels	1	(100.0)	12	(52.2)	12	(66.7)	48	(51.1)	7	(77.8)
2 levels	0	-	3	(13.0)	2	(11.1)	0	-	0	-
Total	1		23		18		94		9	

C: Neonatal mortality rates (per 1,000 live births) by levels of MNH care

	>=30		15 to <30		5 to <15		<5	
7 levels	0	-	1	(3.3)	0	-	0	-
6 levels	0	-	0	-	0	-	1	(1.0)
5 levels	1	(14.3)	3	(10.0)	1	(14.3)	7	(6.9)
4 levels	0	-	5	(16.7)	2	(28.6)	39	(38.6)
3 levels	6	(85.7)	16	(53.3)	4	(57.1)	54	(53.5)
2 levels	0	-	5	(16.7)	0	-	0	-
Total	7		30		7		101	

Note: countries discussed with unspecified numbers of levels are dropped

Table S8: Number of MNH levels (including non-facility) operationalized & recommended across reports

	Facility operationalized		Non-facility operationalized		Facility recommended		Non-facility recommended	
	n	%	n	%	n	%	n	%
Unspecified	55	(26.7)	1	(5.9)	0	-	0	-
7 levels	1	(0.5)	0	-	0	-	0	-
6 levels	1	(5.8)	4	(23.5)	0	-	0	-
5 levels	12	(22.3)	3	(17.6)	1	(11.1)	0	-
4 levels	46	(22.3)	5	(29.4)	3	(33.3)	1	(100.0)
3 levels	83	(40.3)	4	(23.5)	4	(44.4)	0	-
2 levels	8	(3.9)	0	-	1	(11.1)	0	-
Total	206		17		9		1	

Note: "NA" values (papers not discussing numeric facility operationalized levels) dropped

Facility operationalized: Number of levels of care operating in practice, excluding non-facility levels.
Non-facility operationalized: Number of levels of care operating in practice, including non-facility levels.
Facility recommended: Number of levels of care recommended but not implemented, excluding non-facility levels.
Non-facility recommended: Number of levels of care recommended but not implemented, including non-facility levels.
